# Gold Foil

**Published:** 1868-06

**Authors:** 


### Editorial.

### GOLD FOIL.

FEW have a proper appreciation of obtaining and maintaining the proper condition of gold foil, in order to secure the best results in filling teeth. All are aware that the foil of different manufacturers differs, each having its own peculiarity; and foil of the same maker will differ at different times; and these variations all independent of impurities, or rather, existing in gold of absolute purity.

The impairment of foil after its manufacture, by exposure, and by improper methods of using, requires our more immediate attention. Foil to be kept in good condition should be in sealed packages, and close boxes, so that both moisture and dust shall be excluded. In using, the foil should be exposed as short a time as possible.

It should not be brought in contact with the hands, or any thing that would in the least soil it. By contact with the hands it is injured far more than many persons suppose; foil that is quite adhesive may be entirely spoiled, so far as welding is concerned, by having it in contact with the hands for a few moments; this is shown by its failure to unite even under the most skillful manipulation. The contamination is plainly shown by passing a roll of foil that has been manipulated in the fingers through the flame of an alcohol lamp, a smoke is usually quite perceptible, and an offensive odor. Now it requires a heat to expel all of this substance, that injures the foil, for a red heat will often fail to drive it all out of the folds of the roll; and the presence of any particle is objectionable. Always when the adhesive property is to be employed, the foil should be subjected to heat. But to what degree? Certainly not a red heat, at least in a flame. Foil should not be put into a flame, except it is in rather dense rolls or pellets, and then passed so rapidly through as not to become red. The better method is to heat the gold on a metal plate or pan, taking it directly from this, and working it into the filling.

An important item in preparing the foil for filling is to avoid contact with the fingers; to do this, with heavy shears cut the book of foil into two or three parts, then with the pliers lift off the successive leaves of paper and foil, place the latter upon a napkin or piece of linen, a piece of well worn table linen is perhaps the best, this should be folded into a strip about four inches wide and eighteen inches long, having six to eight thicknesses; lay the foil upon this strip, then take in the hand the distant end of the cloth and bring it over upon the foil, and by an adroit backward movement, the foil will be made into a uniformly dense and beautiful roll, any desired density being attainable in a moment. The roll is then taken up with the pliers, and passed through the flame if thought best, but we scarcely regard it necessary, and cut into pieces of the proper size.

We have manipulated foil in this manner for some time, and find it free from objections that always exist when brought in contact with the hands. We are indebted to Dr. C. Palmer for this method, and every time we use it, we feel toward Dr. Palmer, *thank you, Sir.* T.

